# The Simple Rules of Social Contagion

**DOI:** 10.1038/srep04343

**Published:** 2014-03-11

**Authors:** Nathan O. Hodas, Kristina Lerman

**Affiliations:** 1 USC Information Sciences Institute, Marina del Rey, CA 90292

## Abstract

It is commonly believed that information spreads between individuals like a pathogen, with each exposure by an informed friend potentially resulting in a naive individual becoming infected. However, empirical studies of social media suggest that individual response to repeated exposure to information is far more complex. As a proxy for intervention experiments, we compare user responses to multiple exposures on two different social media sites, Twitter and Digg. We show that the position of exposing messages on the user-interface strongly affects social contagion. Accounting for this visibility significantly simplifies the dynamics of social contagion. The likelihood an individual will spread information increases monotonically with exposure, while explicit feedback about how many friends have previously spread it increases the likelihood of a response. We provide a framework for unifying information visibility, divided attention, and explicit social feedback to predict the temporal dynamics of user behavior.

Social media has revolutionized how people create and consume information. Unlike the broadcasts of traditional media, which are passively consumed, social media depends on users to deliberately propagate the information they receive to their social contacts. This process, called social contagion, can amplify the spread of information in a social network. Understanding the mechanics of social contagion is crucial to many applications: creating viral marketing campaigns, evaluating the quality of information, and predicting how far it will spread. While the spread of information is often likened to an infectious disease^1^,^2^,^3^,^4^, social contagion differs in that social media users actively seek out information and consciously decide to propagate it. Because of the constraints of available time and cognitive resources, the ease of discovery will significantly affect information's propensity to go viral^5^,^6^. The enormous flux of available social media content often saturates user's ability to process information. In most studies of information propagation on networks, users are considered exposed if they received a message, regardless of whether they see it or not, which can lead to counterintuitive results suggesting that additional exposures inhibit response^7^,^8^. In reality, of a user seeing a message depends on how the website arranges content, the flux of incoming information, and the effort the user is willing to expend in discovering information. By accounting for these factors, we demonstrate that social contagion is quite simple and people's responses can be accurately predicted.

From a theoretical perspective, one of the simplest and most widely studied models of social contagion is the independent cascade model (ICM)^1^,^2^,^3^,^4^,^9^,^10^. The ICM-class of models assume that each exposure of a healthy (naive) person by an infected (informed) friend leads to an independent chance of information transmission. Therefore, the probability that a healthy individual becomes infected increases monotonically with the number of exposures, potentially causing a global epidemic involving a substantial fraction of the population^11^,^12^. However, studies of information spread in social media have identified social behaviors that qualitatively differ from predictions of the ICM. For example, when measuring how people respond to their friends' use of certain memes or recommendations for news articles, repeated exposure initially increases infection probability, but eventually exposure appears to be inhibitory^7^,^8^, violating the central assumptions of the ICM. A number of explanations have been offered for this aberration, including complex contagion^13^,^14^,^15^. In complex contagion, the probability to adopt a behavior, or an idea, varies with the extent of exposure, suggesting that social phenomena may drive response and interact non-trivially with network structure^16^,^17^,^18^. An alternative explanation invokes the linear threshold model, in which the proportion of friends (past a certain threshold) adopting a behavior determines contagion^2^,^19^,^20^. Among other factors thought to affect social contagion are the novelty^21^ or persistence^7^ of information, and competition with other information^6^. The role of cognitive constraints in online social interactions has not been widely examined, although one study of Twitter demonstrated that people limit themselves to approximately 150 conversation partners^22^, a number similar to the bound on human social group size^23^.

To compare how visibility and social factors contribute to contagion, we collected data from two online social networks: Digg and Twitter. The microblogging service Twitter allows registered users to broadcast short messages, called tweets, to their followers. A message may contain a URL to external web content. In addition to posting a new message, a user can also retweet an existing message, analogous to forwarding an email. Twitter users create social links by following other users. Each link is directed: we refer to the followed user as the friend, and the following user as the follower. Upon visiting Twitter, a user is presented with a list containing tweets made by friends, with the most recent tweet (or retweet) at the top.

Social news aggregator Digg leverages opinions of its users to help people discover interesting news stories. Users submit URLs to news stories and vote for, or *digg*, stories submitted by others. Users can follow the activity of others. The social user-interface on Digg shows a user a stream of stories his or her friends recently submitted or voted for. The stream is ordered chronologically by time of earliest recommendation (submission or vote) by a friend, with the most recent newly-recommended story at the top. When a user votes for a story, the recommendation is broadcast to a user's followers. However, additional recommendations do not change the story's relative position in the user's default social stream. Instead, a badge appears next to the story showing how many friends have recommended it. When the story receives enough votes, Digg promotes it to its front page. However, before promotion, it can be found through friends' recommendations or on the newly submitted stories list, which at the time of data collection was receiving tens of thousands of new submissions daily.

We use techniques, originating from non-equilibrium statistical physics, to analyze user behavior on these sites. Our approach enables us to separate the factors of social contagion that are attributable to the visibility of information (i.e., how easily it can be discovered in the user interface of each site) from the factors attributable to social influence. After accounting for these factors, social contagion becomes quite simple: each exposure increases the likelihood of a response, and social signals about the number of friends who have previously adopted the information (when such signals are provided by the web site) further amplify response. We demonstrate that we are able to accurately forecast an individual's behavior in real-time on both sites.

## Results

**Figure 1:**
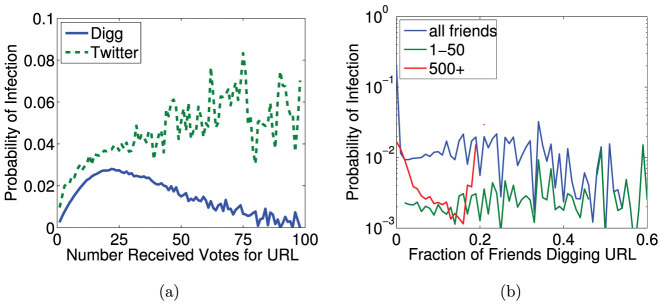
The exposure response functions for Twitter and Digg, (a) as a function of total number of votes for the URL a user receives in their information stream, and (b) as a function of the fraction of friends adopting story, for Digg only. The equivalent results for Twitter can be found in^5^.

**Figure 2:**
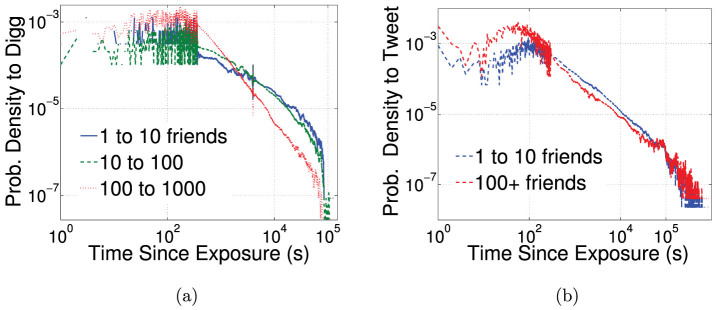
The time response functions for (a) Digg and (b) Twitter for different user classes.

Using URLs as markers, we study the spread of information through the follower graphs of Digg and Twitter. A user may be exposed multiple times by friends to a URL. The exposure response function gives the probability of an infection as a function of the number of such exposures. An exposure is defined to occur when a message containing the URL arrives in the user's stream, even if the user does not consciously see it. When aggregated over all users, both Twitter and Digg exposure response functions suggest complex contagion^7^: while initial exposures increase infection probability, further exposures appear to saturate (Twitter) or suppress (Digg) further infection (Fig. 1a). Aggregated exposure response obscures heterogeneous behavior, because it conflates the response of users with different cognitive loads, i.e., different quantities of information in their stream. A large volume of incoming information, which scales with the number of friends a user follows as 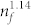, reduces the user's ability to find any specific message^24^,^25^. The likelihood a user will find a message containing the URL depends on *n_f_*, denoted 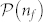
5. However, disaggregating only partially ameliorates complications due to underlying heterogeneity; although plotting infection as a function of the fraction of friends adopting the URL on Twitter displays remarkable consistence between user groups (Fig. 2 in^5^), a similar plot using Digg data (Fig. 1b) suggests the contradictory and confusing result that even small increases in exposure dramatically suppress infection. Although a linear threshold model may be consistent with Fig. 1a, neither the ICM nor linear threshold model can simultaneously account for observed trends on Twitter and Digg.

To resolve this contradiction, consider the process of infection on each site. To become infected, a user must first discover at least one message containing the URL. The likelihood the user will see a specific message depends on its position in the user's stream. We use ‘visibility' to refer to this quantity. A new message starts at the top of the queue, where it is more likely to be seen because users usually start browsing from the top of a page^26^. With time, newer messages push it down the queue, where a user is less likely to see it^27^,^28^. We approximate a message's dynamic visibility using the time response function, 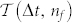, the probability that a user with *n_f_* friends retweets or votes at a time Δ*t* after the exposure^5^. We plot 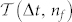 for Digg and Twitter in Fig. 2a and 2b, respectively, demonstrating that the visibility of a new message decays rapidly in time. Digg stories were only followed until promotion, which occurs at most 24 hours after appearing on Digg. The data are smoothed using progressively wider smoothing windows, as in^5^.

A model describing user response to multiple exposures must consider the visibility of each exposure. In addition, a website's use of any social signals — for example, displaying the number of friends who recommended the URL — may alter user response, given that they have found the URL. The probability that a user with *n_f_* friends will be infected after *n_e_* exposures is 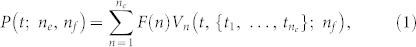
where *V_n_*() is the probability of finding *n* of the *n_e_* exposures occurring at the times 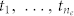, and *F*(*n*) is the social enhancement factor accounting for the user observing that *n* of their friends have recommended the story. Note that this formalism averages out content-specific factors and variable weights that a user may ascribe to different friends.

The particular functional form of *V_n_* depends on details of the website user-interface. On Twitter, all messages start at the top of the stream. By scanning the stream, a user can discover each message independently, so any of the exposures can result in an infection. This behavior is well approximated by the probability of becoming infected by at least one exposure (see Supplement), given by 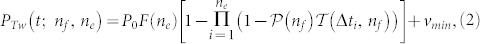
where *v_min_* is the effective minimum visibility of a message in the Twitter interface, the proportionality *P*_0_ is fitted by minimizing weighted mean absolute percent (WMAP), as described in the Methods, and *n_e_* is the number of exposures to the URL at time *t*. Underlying activity rates and cultural norms vary from site to site, so *P*_0_ can be interpreted as a task-specific scale factor. The constant *v_min_* is due to the ability to discover the URL outside the social media site or via other interfaces.

We calculate 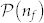 by measuring the average probability of retweeting the URL for users who were exposed only once. The average is taken over all users with *n_f_* friends, as described in^5^,^25^. The time response function 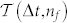 describes the visibility after Δ*t* seconds since exposure. Specifically, it is the probability that a user with *n_f_* friends retweets/votes at the indicated interval Δ*t* after a URL's arrival, given that the user votes on that URL.

**Figure 3:**
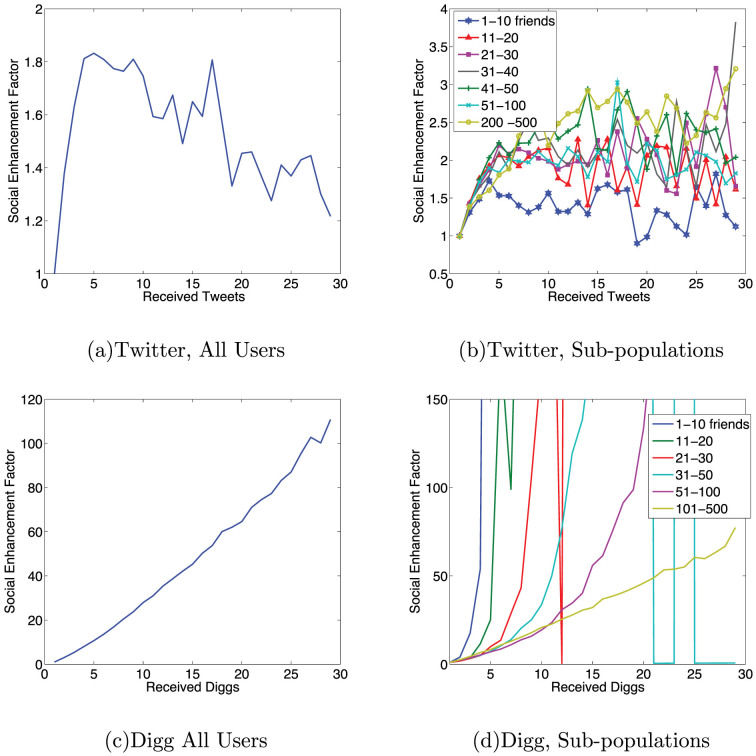
The social enhancement factors for Twitter and Digg. (a,c) Averaged over all users, (b,d) Calculated for sub-populations based on *n_f_*. The decay in the social enhancement factor for Twitter can be attributed to residual spam in the dataset.

The Digg user-interface differs from Twitter in that messages are by default ordered by the time of their *first* appearance in the user's stream. Additional votes do not alter its position but are reflected in a badge next to the URL showing the number of friends, *n_e_*, who voted for the URL. The badge provides a social signal, which may alter user response. Because of the user-interface, Eq. (1) reduces to 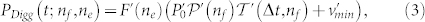
where Δ*t* is the time elapsed from the first vote by a friend, and the primes indicate Digg specific values for each quantity. We empirically determined *F*′(*n_e_*) using a maximum likelihood estimate, described in the Methods. Social feedback in Digg results in large amplification of the probability of infection, shown in Fig. 3c. This could have multiple origins, including endorsement by friends^29^, or from the increased visibility of the URL via alternative ways of discovering it on Digg, such as sorting URLs by popularity.

**Figure 4:**
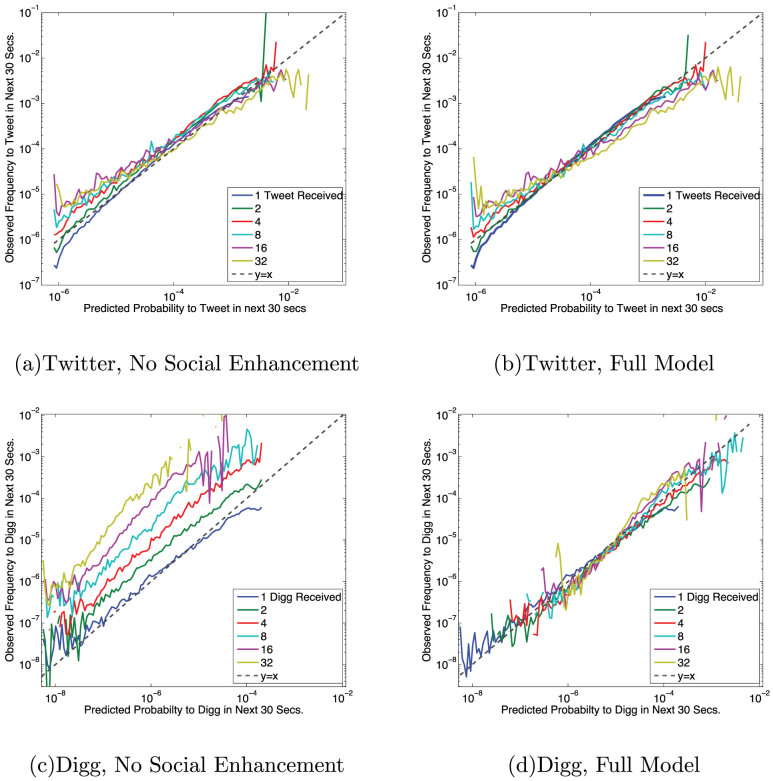
Forecasting accuracy for Twitter and Digg.

To validate the proposed model of social contagion, we forecast user activity and compare it to observed activity. Specifically, we calculate the observed frequency that a user with *n_f_* friends retweeted a URL in our Twitter dataset or voted for one in the Digg dataset in the subsequent 30 seconds. Then, using Eq. (2) or Eq. (3), we calculate the theoretical probability that a user with that many friends would act in those 30 seconds, given the same exposures. Data were divided into a test set and training set. Parameters were estimated on the training set. Results are shown from the test set. Plotting the predicted versus observed probabilities allows us to graphically assess the accuracy of the contagion model. Unbiased forecasts lie along the unit-slope line. The forecasted responses on Twitter (Fig. 4b) and Digg (Fig. 4d) have a WMAP error of 0.5% and 1.5%, respectively. Ignoring social enhancement, and thereby utilizing ICM, produces systematically biased results, shown in Figs. 4a and 4c. Without this social enhancement, Twitter and Digg have WMAP error of 0.7% and 12.2%, respectively. Although we do not know the specific cause of this difference, we may surmise that it is due primarily to the explicit social feedback present on Digg but absent on Twitter. It appears users on Twitter adopted content based primarily on ease of discovery (visibility). Additionally, a model not incorporating visibility decay could not account for variations in user-interface, i.e., Eqs. (2) and (3).

The unbiased fidelity of the proposed model suggests that once visibility of the exposures is taken into account, social contagion operates as a simple contagion, i.e., with infection probability increasing monotonically with the number of exposures. Complex contagion, where “network effects,” appear to play a significant role in the contagion process, may to a large extent be due to the combined factors of visibility and direct social enhancement factors. Moreover, by comparing two different websites with very different user-interfaces, we have demonstrated that it is possible to isolate the factors in social contagion due to social feedback and the user-interface, without directly manipulating the underlying social network or user-interface^29^,^30^.

Rapid visibility decay, combined with decreased susceptibility of highly connected users, explains why information in social media fails to spread as widely as predicted by the generic ICM^8^. Although different types of information may spread according to slightly different patterns^31^,^32^ our analysis is content agnostic, so the reported results are the population average. Explicit social feedback can significantly magnify user response, albeit making it less useful for popularizing high-quality content^33^. Unlike Digg, the Twitter user-interface offered no explicit social feedback (beyond trending topics). Users may remember seeing a friend's recommendation of the URL, a factor that could explain the slight social enhancement seen in Twitter response in Fig. 3a. When explicit social feedback is available, as in Digg, Fig. 3d shows that users appear to weigh their actions based on the fraction of friends endorsing a URL instead of considering the absolute number.

## Discussion

We show that there are important and surprising differences between the diffusion of information and a disease stemming from cognitive limitations for processing information. In pathogenic contagion^34^, people with more incoming contacts are more likely to contract a disease, but in social contagion such people are less likely to become infected: because the volume of information scales with the number of friends a user follows, highly connected users are less likely to notice a particular piece of information, and they require stronger social signals to act (Fig. 3d), on average, than poorly connected users^5^,^25^. These highly connected users dominate the high-exposure portion of the average exposure response function (Fig. 1), giving the false impression that more exposures may be counter-productive. Granted, highly connected users tend to be infected earlier^35^ and also to have more followers^24^, increasing their influence once they are infected^17^,^35^. Users in a tightly connected core of friends may be repeatedly exposed to information, and the present work demonstrates how the combination of social enhancement and awareness contribute to the observed behavior of users in high k-cores participating in larger cascades^17^.

By comparing the dynamics of two different websites, we have demonstrated that it is possible to isolate factors in social contagion due to social feedback and the user-interface, without manipulating the underlying social network or user-interface^16^,^29^,^30^. Moreover, the unbiased fidelity of our model suggests that once visibility of the exposures is taken into account, social contagion operates as a simple contagion, i.e., with infection probability increasing monotonically with the number of exposures. Although our forecasts are only for action within the subsequent 30 seconds, the present work shows that this near-term likelihood can vary by over a factor of 10,000. Although longer periods could be forecast, intervening events, such as receiving additional messages, would invalidate the initial conditions of the forecast. This could be corrected by utilizing higher-order models accounting for the probability of additional messages being received during the forecast window.

Our work highlights how cognitive constraints impact digital content sharing activities. Humans have developed large brains, partly to handle the mental demands of social life^36^,^37^, but constraints imposed by our brain's finite information processing bandwidth affect social behavior, for example, by limiting maximum group size^23^. Our present results suggest cognitive constraints also affect how individuals utilize information in their dynamic social media streams. Attentive acts, such as browsing a website and reading tweets, require energy; because the brain's capacity for mental effort is limited by its energy requirements, so is our attention^38^. This will reduce responsiveness under conditions of high information load, making explicit social feedback essential for determining the allocation of cognitive resources. Thus, social contagion will be highly dependent on explicit social feedback and the user-interface.

Implicit in our work is the utilization of Big Data as a microscope: we uncover behavioral mechanisms and even the difference between user interfaces. Regardless of the social synergy desired by the website, information discovery costs appear to be an important factor in determining accuracy of activity forecasts. The site's design choice regarding its visibility policy will largely determine the quality of the user experience regarding information discovery and spread. Digg does not refresh the position of information after each recommendation, and the social signals it uses do not compensate for the loss of visibility it suffers over time. Although the current work provides techniques for real-time forecasting of the average user behavior on a specific website, understanding the emergence of globe-spanning viral content will require accounting for the interaction of the dynamic visibility and social synergy across a multitude of websites and media outlets.

## Methods

### Data sources

We used Twitter's Gardenhose API to collect tweets over three weeks in Fall of 2010. We retained tweets containing a URL in the message body. We used Twitter's search API to retrieve all tweets containing those URLs, ensuring the complete tweeting history of all URLs, resulting in 3 million tweets in total. We also collected the friend and follower information for all tweeting users, resulting in a social graph with almost 700 K nodes and over 36 M edges. We removed URLs whose retweeting behavior exhibits patterns associated with spam or automatic activity^39^, leaving us a data set containing 2 K distinct URL's retweeted a total of 213 K times. We use time stamps in tweet metadata combined with the follower graph to track when users are exposed to URLs by a friend and when they retweet them. We define a retweet to be anytime a user tweets a URL that had previously appeared in her Twitter feed. We did not resolve link-shorteners, so different URLs might map to identical content, but we considered each URL to be a unique marker of information. After removing spam URLs, we only consider events where users received a particular URL less than 30 times, to further eliminate likely spam URLs.

We used the Digg API to collect data about 3.5 K stories promoted to the front page in June 2009 and the times at which 140 K distinct users voted for these stories. We also collected information about voters' friends, giving us a social graph with 280 K users and 1.7 M links. For the present analysis, unless noted otherwise we consider only the voting dynamics occurring before promotion to the front page, so the primary means of information propagation is through the friends interface. Both datasets were divided into training and test sets to rule-out over-fitting in determining the correct interpretation of the data.

To calculate probabilities of response to multiple exposures, the data was broken down into separate time series, each corresponding to the arrival of specific URL-containing tweets or votes into a single user's stream. For each series, at every one-second interval we calculate the quantity we define as ‘visibility' of the URL: 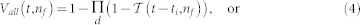

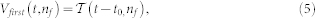
where *n_f_* is the number of friends of the user, 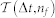 is the time response function for a user with *n_f_* friends. *V_all_* is proportional to the probability of finding any one of the received messages at time *t*, while *V_first_* is proportional to the probability of finding only the first message.

### Data analysis

We calculate 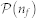 by measuring the average probability of retweeting the URL for users who were exposed once and only once to it. The average is taken over all users with *n_f_* friends, as described in^5^,^25^. The time response function 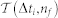 describes the visibility of a message since exposure at *t_i_*. This is given by probability, shown in Fig. 2, that a user with *n_f_* friends will retweet a time Δ*t_i_* after the exposure, given that retweeting occurred.

The time response function, 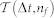 is produced by calculating, using the observed data, the probability that a user retweets/votes at the indicated interval Δ*t* after a URL's arrival, given that the user votes on that URL. For Twitter data we calculate the time response function only for those events in which a user received the URL once and only once. For Digg, this constraint is lifted, because there are too few such events in the Digg data. The precise time response function depends on *n_f_*, because users with many friends receive new messages at a higher rate, causing the visibility of any specific message to decay more quickly^5^. We lack sufficient data to precisely calculate the time response function for each *n_f_*. Instead, we calculated the time response function for users with *n_f_* = 1–2, *n_f_* = 9–11, and *n_f_* = 90–110, producing 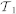, 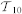, and 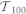, respectively, following the procedure in^5^. To estimate the time response function for arbitrary *n_f_*, we interpolated as follows: 
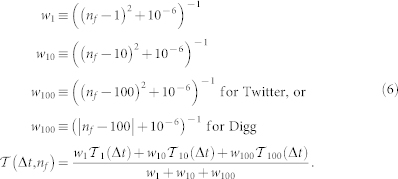
To produce the fits for *v_min_* and *P*_0_, we plot the theoretical probability versus the observed probability for an event observed in the data, i.e. forming a function *O*(*p*), where *p* is the calculated probability. That is, no numerical simulations were used, but event timings and the follower network were taken directly from the observed data. We isolated the events corresponding to a receiving a single message, leading to a subset of predictions denoted *O*_1_(*p*). We then minimize the weighted mean absolute percent error (WMAP)^40^, 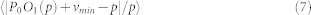
by searching over *P*_0_ and *v_min_*. For Digg, we have *P*_0_ = 667, log(*v_min_*) = −19. An analytical form for 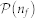 was determined by fitting to minimize RMS error of the empirically determined 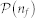
5, giving Digg's 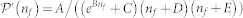, where *A* = 7.6 · 10^−3^, *B* = −6.2 · 10^−2^, *C* = 1.7 · 10^−3^, *D* = 3.7, *E* = 17.8. For Twitter we have *P*_0_ = 16.6 and log(*v_min_*) = −14, and we used 
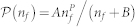
, where *A* = 0.3, *P* = 0.16, *C* = 0.55. Note the *E* was chosen by minimizing *WMAP* error simultaneously with fitting *P*_0_ and *v_min_* on the training data, i.e. *E*'s purpose is to correct for sparsity in the empirically calculated 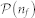 for Digg.

To calculate the social enhancement factors, we carry out the MLE for *F*(*n_e_*) in the following manner. We take as axiomatic the true probability of a response given *n_e_* exposures is *F*(*n_e_*)*P*(*υ*), where *υ* parameterizes the underlying visibility. Thus, given *N*(*υ*) observed events for a specific *υ*, the likelihood, 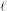, of observing *N_r_*(*υ*) responses is determined by the binomial distribution 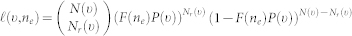. The total log-likelihood of observing the curve 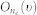 is thus 
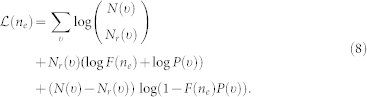
For each value of *n_e_*, we find the value of *F*(*n_e_*) that maximizes 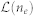. First, for *n_e_* = 1, we define *F*(1) = 1, so we obtain the MLE for *P*(*υ*) using 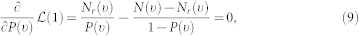
giving *P*(*υ*) = *N_r_*(*υ*)/*N*(*υ*). Then, for *n_e_* > 1, we are left to find the likelihood maximizing *F*(*n_e_*) given *P*(*υ*), leading to 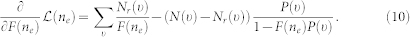
Numerically solving for 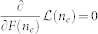 provides the MLE for *F*(*n_e_*).

The minimum possible observed probability is bounded by the number of observed events. In the forecasting predictions, the friend-cohort breakdown in Fig. 4 appears to deviate from the observed probabilities at very high and low predicted probabilities. However, this is due to the minimum probability floor rising beyond the predicted = observed line, because events with high visibility and high social influence or very low visibility are less common.

## Author Contributions

N.H. and K.L. developed the model. N.H. performed empirical analysis and evaluation. Both authors contributed to the manuscript.

## Supplementary Material

Supplementary Information
