# Supplementary material for: The Simple Rules of Social Contagion
Source: Sci Rep. 2014 Mar 11;4:4343. doi: 10.1038/srep04343 (PMC3949249; doi:10.1038/srep04343)
Supplement: Supplementary Information [file srep04343-s1.pdf]

Supplementary Material

The Simple Rules of Social Contagion

Nathan O. Hodas and Kristina Lerman

Approximating Visibility Functions

Depending on the user-interface, the user may be exposed to a URL from a variety of different messages. Under general conditions, the probability of discovering a URL will depend on the visibility of each of those messages. In addition, the user's response may differ depending on how many times they actually observed the URL. Thus, the probability of being infected by a URL will depend on the probability of seeing the URL n times and an enhancement factor, $f(n; n_f)$, arising from the collective effect of multiple exposures. The probability of acting at time t is, therefore,

$$P(t, n_e; n_f) = \sum_{n=1}^{n_e} f(n; n_f) V_n(t, \{t_1, \ldots, t_{n_e}\}; n_f),$$

where $V_n(t, \{t_1, \ldots, t_n\}; n_f)$ is the probability of explicitly observing n of the n_e URL's that arrived at times $t_1, \ldots,\ t_{n_e}$. For Digg before promotion to the front page, the URL is ordered by the time of its first recommendation to the user, so $V_n(t) = \delta_{n,n_e} \mathcal{P}(n_f) \mathcal{T}(n, n_f)$, where δ_{n,n_e} is the Kronecker delta function. Thus, only one term is relevant for Digg. Approximating $f(n_e; n_f)$ as the social enhancement factor $F(n_e)$ gives the probability of Digging a URL a user receives to be Eq. (3).

For Twitter, each tweet is displayed based on the chronological order of its arrival, so there may be multiple tweets potentially containing the same URL in the user's stream. Each tweet decays in visibility according to the time-response function, based on the time of its arrival in the user's stream. Thus, the probability of discovering tweet i containing the URL arriving at time t_i is $\mathcal{P}(n_f)\mathcal{T}(t - t_i, n_f)$. For brevity, we will abbreviate this quantity as $\tau_i \equiv \mathcal{P}(n_f)\mathcal{T}(t - t_i, n_f)$. The probability of seeing a URL only once is

$$V_1(t) = \sum_{i}^{n_e} \prod_{j \neq i}^{n_e} \tau_i (1 - \tau_j).$$

Similarly, the probability of seeing exactly two out of n_e URL's is

$$V_2(t) = \sum_{i}^{n_e - 1} \sum_{j > i}^{n_e} \prod_{j \neq i}^{n_e} \prod_{k \neq i,j}^{n_e} \tau_i \tau_j (1 - \tau_k).$$

As n_e grows, the number of combinations required to enumerate each V_n grows rapidly with n_e. V_n will have $n_e!/n!(n_e-n)!$ terms. Although one could calculate all V_n explicitly every time-step for every user and URL, this is currently computationally prohibitive. We propose an approximate form for P_{Tw}, Eq. (2), justified as follows.

Although each V_n for Twitter may have many terms, it can be represented succinctly using a generation function,

$$V_n = \mathbb{C}_{n_e}\frac{1}{n!}\frac{\partial^n}{\partial y^n}\prod_{i=1}^{n_e}\left(1+\frac{\tau_i}{1-\tau_i}y\right)\Bigg|_{y=0},$$

where $\mathbb{C}_{n_e} \equiv \prod_{i=1}^{n_e}(1-\tau_i)$ is the probability of not seeing any of the n_e tweets. Using this form, the exact expression for P_{Tw} is

$$P_{exact} = \mathbb{C}_{n_e}\sum_{n=1}^{n_e}\frac{f(n;n_f)}{n!}\frac{\partial^n}{\partial y^n}\prod_{i=1}^{n_e}\left(1+\frac{\tau_i}{1-\tau_i}y\right)\Bigg|_{y=0}.$$

We wish to know how well P_{exact} can be approximated if we take $f(n;n_f) = F_{tw}(n_e)$, i.e. determining the probability of seeing any URL, with a social enhancement factor. Using generating functions, this approximation is expressed as

$$\begin{aligned} P^* =& \mathbb{C}_{n_e}F_{tw}(n_e)\sum_{n=1}^{n_e}\frac{1}{n!}\frac{\partial^n}{\partial y^n}\prod_{i=1}^{n_e}\left(1+\frac{\tau_i}{1-\tau_i}y\right)\Bigg|_{y=0} \\ =& \mathbb{C}_{n_e}F_{tw}(n_e)\left(e^{\frac{\partial}{\partial y}}-1\right)\prod_{i=1}^{n_e}\left(1+\frac{\tau_i}{1-\tau_i}y\right)\Bigg|_{y=0}, \end{aligned}$$

where $e^{\frac{\partial}{\partial y}} = \sum_{n=0}\frac{1}{n!}\frac{\partial^n}{\partial y^n}$. One may show that $e^{a\frac{\partial}{\partial y}}f(y) = f(y+a)$ by left-multiplying both sides of this identity by the inverse operator $e^{-a\frac{\partial}{\partial y}}$. Using this identity gives

$$\begin{aligned} P^* =& \mathbb{C}_{n_e}F_{tw}(n_e)\left(\prod_{i=1}^{n_e}(1+\frac{\tau_i}{1-\tau_i})-1\right) \\ =& F_{tw}(n_e)\left(1-\prod_{i=1}^{n_e}(1-\mathcal{P}(n_f)\mathcal{T}(\Delta t_i,n_f))\right), \end{aligned}$$

where we have expanded all of the definitions in the last line above. To find the best choice for $F_{tw}(n_e)$ to determine its suitability as an approximation, we define the ratio

$$F^*(n_e,t) \equiv \frac{P_{exact}}{P^*/F_{tw}(n_e)} = \frac{\hat{S}_f G(y)}{\left(e^{\frac{\partial}{\partial y}}-1\right)G(y)}\Bigg|_{y=0},$$

where $\hat{S}_f \equiv \sum_{n=1}^{n_e}\frac{f(n;n_f)}{n!}\frac{\partial^n}{\partial y^n}$ and $G(y) \equiv \prod_{i=1}^{n_e}\left(1+\frac{\tau_i}{1-\tau_i}y\right)$. That is, $F^*(n_e,t)$ is simply the time-dependent ratio of the exact expression for seeing one of n_e tweets to the approximated

form (without $F_{tw}(n_e)$). If this quantity varies very little with time, then taking $F^*(n_e,t) \sim F_{tw}(n_e)$ will give a good approximation.

Because of the nature of the Digg interface, as stated above, we can directly observe plausible forms for the $f(n;n_f)$ enhancements. We observe that Digg enhancements are generally linear, and we may surmise an approximate form for the Twitter enhancements to be $f(n;n_f) \approx \alpha n + \beta$. This is not to hypothesize a true form of $f(n)$ for Twitter but to merely provide a plausible function form to test the accuracy of the proposed approximation. This gives

$$\begin{aligned}\hat{S}_f =&\alpha \sum_{n=1}^{n_e} \frac{n}{n!}\frac{\partial^n}{\partial y^n} + \beta \left(e^{\frac{\partial}{\partial y}} - 1\right) \\ =&\alpha \frac{\partial}{\partial y} e^{\frac{\partial}{\partial y}} + \beta \left(e^{\frac{\partial}{\partial y}} - 1\right).\end{aligned}$$

Replacing the first sum is possible because derivative-orders greater than n_e evaluate to 0. Returning to the expression for $F^*(n_e,t)$, we have

$$\begin{aligned}F^*(n_e,t) &= \frac{\alpha G'(1) + \beta(G(1)-1)}{G(1)-1} \\ &= \alpha \frac{\sum_{i=1}^{n_3} \tau_i}{1 - \prod_{i=1}^{n_e}(1-\tau_i)} + \beta.\end{aligned}$$

Because each τ is proportional to the time-response function, F^*_{tw} varies with time. Because the probabilities of conducting any action on Twitter at any instant are low, $\tau \ll 1$. Consider the two extreme, yet plausible, scenarios: 1) The user receives n_e messages simultaneously all with maximum visibility or 2) The user receives n_e messages which have decayed to extremely low visibility, so $\tau \to v_{min}$. For case 1, the maximum visibility corresponds to $\mathcal{P}(n_f)\mathcal{T}(\sim 0, n_f)$, which is very small, i.e., $< 10^-3$ [7]. For case 2, the minimum visibility is v_{min}, so for either limit we have

$$F^* = \alpha n_e \tau_0 / \left(1 - (1-\tau_0)^{n_e}\right) + \beta \approx \alpha + \beta + \frac{\alpha}{2}(n_e - 1)\tau_0.$$

In case 1, $\tau_0 = \tau_{max}$, and in case 2, $\tau_0 = v_{min}$. Thus, in either limit, F^* will tend to have a characteristic value of $\alpha + \beta$, varying weakly with time, confirming the argument that the full P_{exact} can be approximated by P^*.
